# Supplementary material for: Molecular Characterization and Expression Profile of PaCOL1, a CONSTANS-like Gene in Phalaenopsis Orchid
Source: Plants (Basel). 2020 Jan 4;9(1):68. doi: 10.3390/plants9010068 (PMC7020484; doi:10.3390/plants9010068)
Supplement: Supplementary file 1 [file plants-09-00068-s001.zip › Yeh/Supplementary Figures_103.docx]

**Supplementary Figures:**


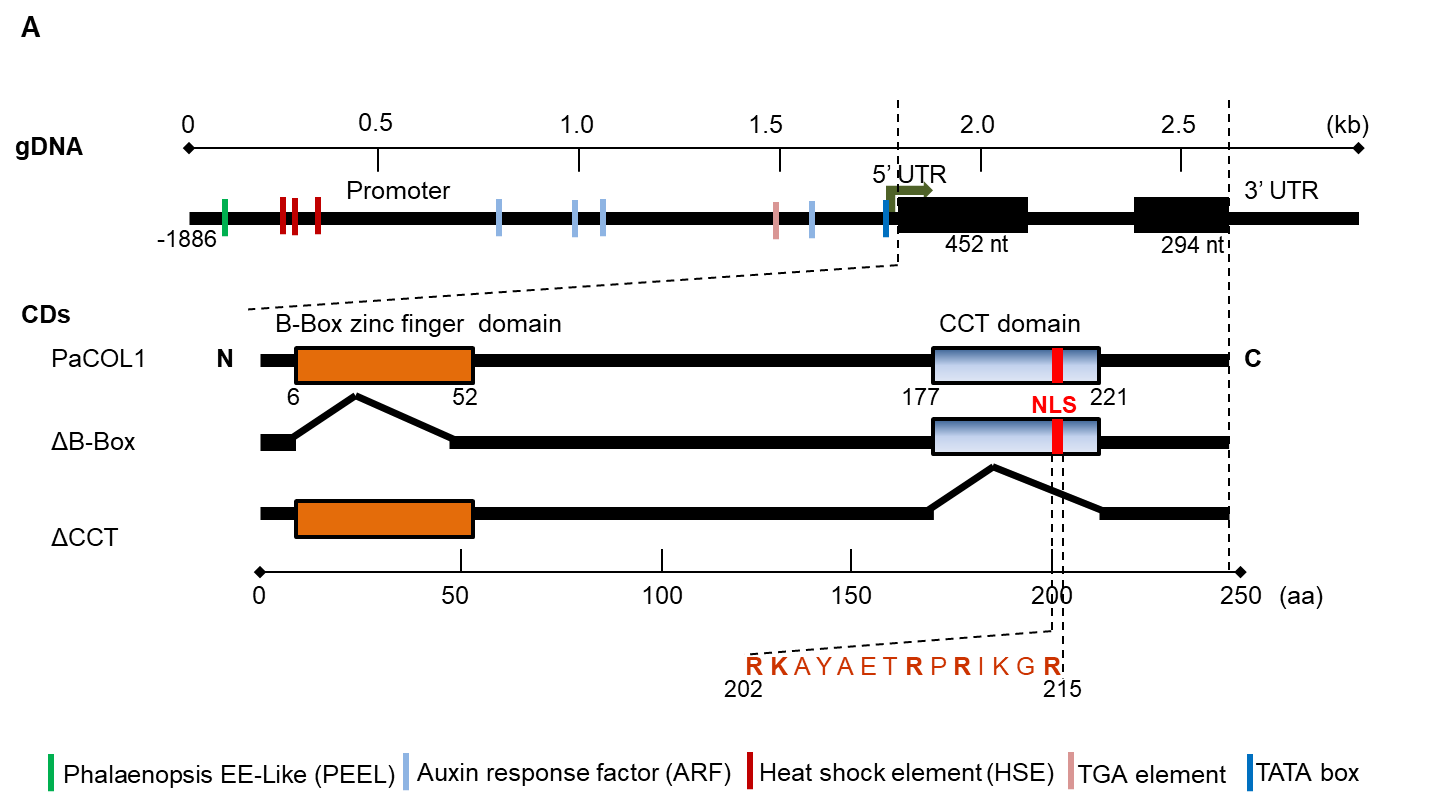


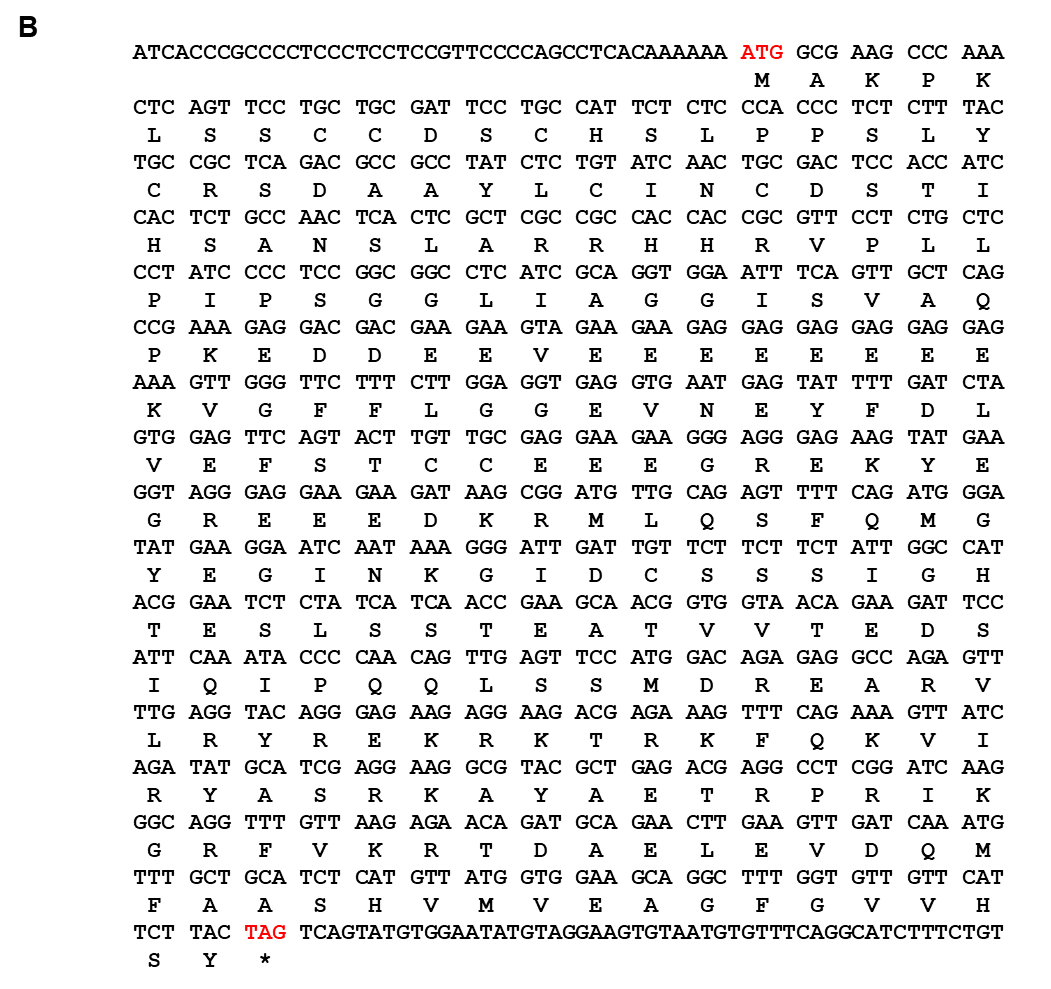


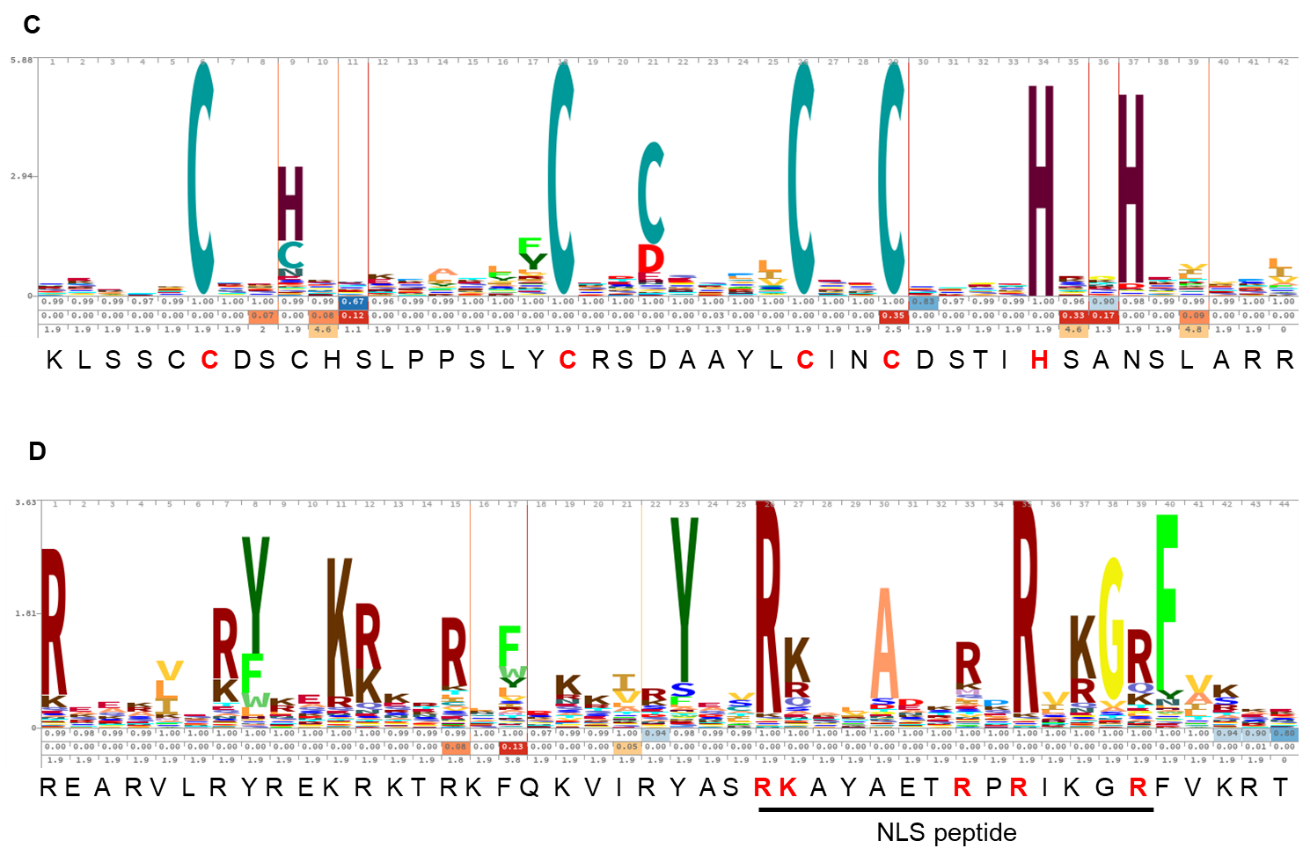
**Figure S1**: Gene structure of PaCOL1. (**A**) The sequence structures of PaCOL1 are illustrated in scale. The conserved sequence of NLS and elements used to tests are also listed. PaCOL1, the full length of PaCOL1. ∆B-Box, PaCOL1 gene deleted B-Box domain sequences. ∆CCT, PaCOL1 gene delete CCT motif sequences. (**B**) The full-length cDNA sequence and deduced amino acid sequence of PaCOL1 were analyzed by using the translate program in the ExPASy Bioinformatics Resource Portal. (**C**) The consensus sequence of the B-box domain (PF00643) and (**D**) CCT motif (PF06203) of the BBX protein family from the Pfam database. The sequences from PaCOL1 are shown below. The highly conserved amino acids are in red.


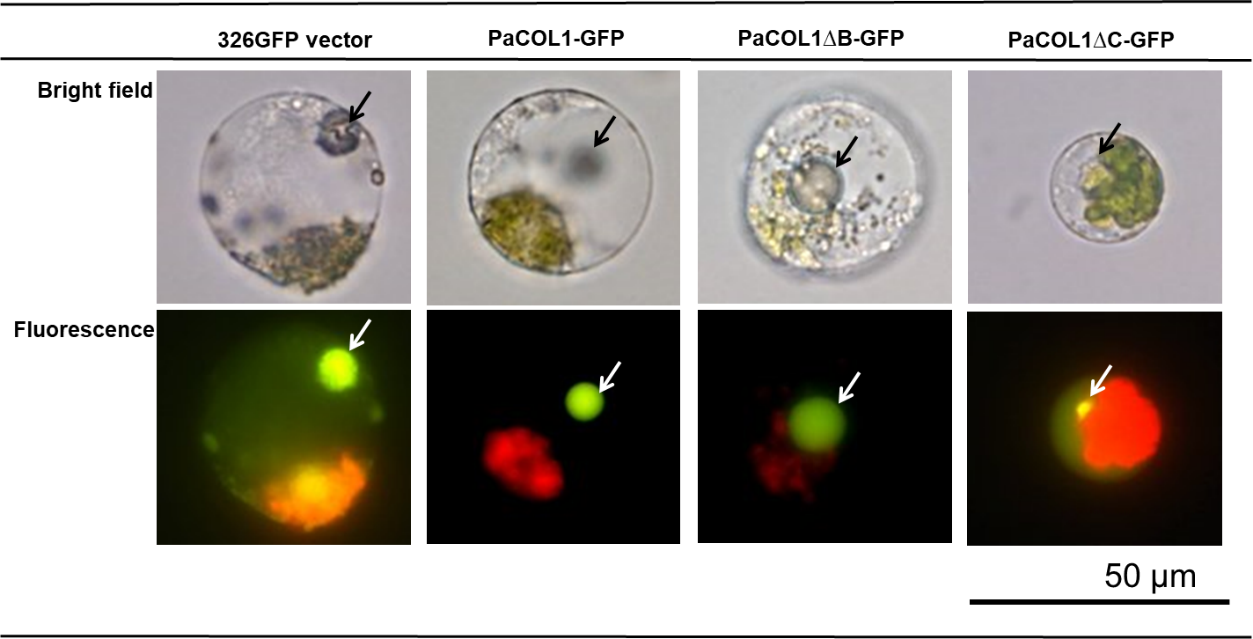


**Figure S2**: Subcellular localization of PaCOL1 and 2 truncated mutant proteins in orchid protoplasts visualized by fluorescence microscopy. Orchid protoplasts were transformed with PaCOL1, PaCOL1ΔB, and PaCOL1ΔC fusion constructs. The arrow indicates cell nuclei. Scale bar = 50 µm.


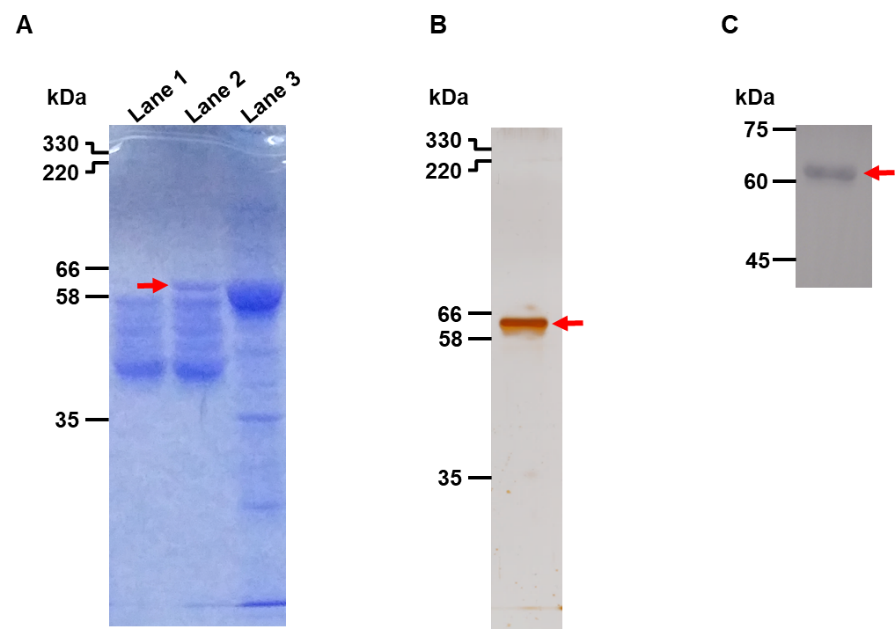


**Figure S3**: Fusion protein purification and identification for antibody production. (**A**) The coomassie blue stained SDS-PAGE of extract and purified the fusion protein from E. coli BL21 strain with different concentrations of Triton X-100. Lane 1 and lane 2, the sample contained 1% and 5% Triton X-100 in breaking buffer. Lane 3, the sample collected after IPTG induction. (**B**) The 0.5-µg sample of molecular weight 60.2-kDa samples after SDS-PAGE gel extraction checked by silver staining. (**C**) Before specific antibody processing, fusion proteins were recognized by using a commercial MBP-tag antibody.


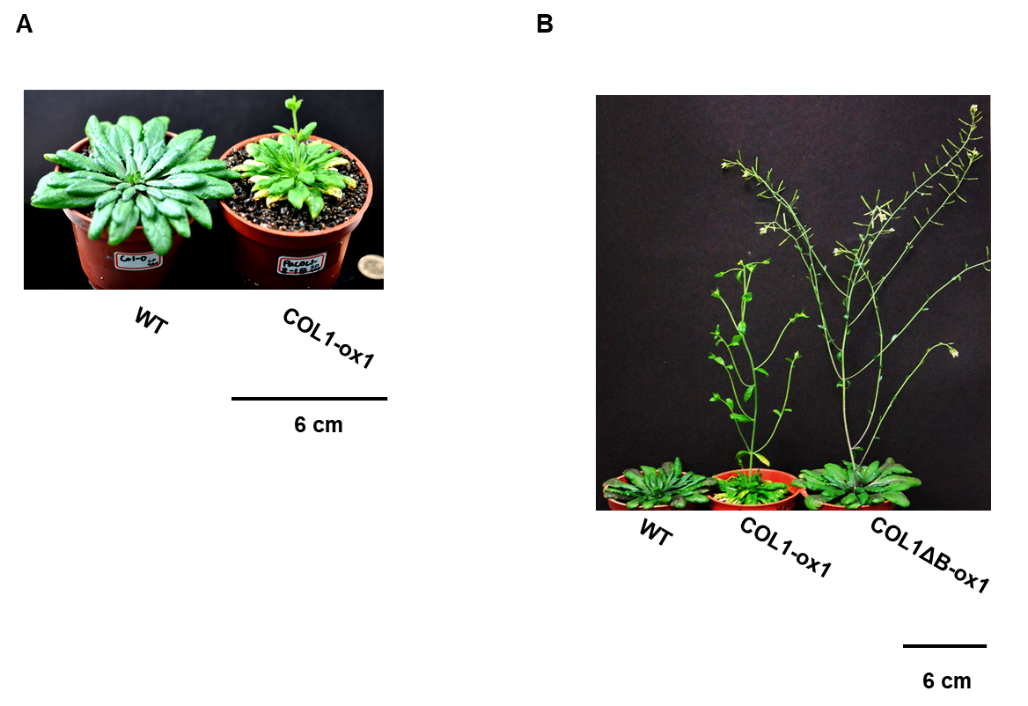


**Figure S4**: The phenotypes of PaCOL1 transgenic plants and the WT. (**A**) 60-day-old plants of WT and COL1-ox1. (**B**) 80-day-old plant of WT, COL1-ox and COL1∆B-ox plants.


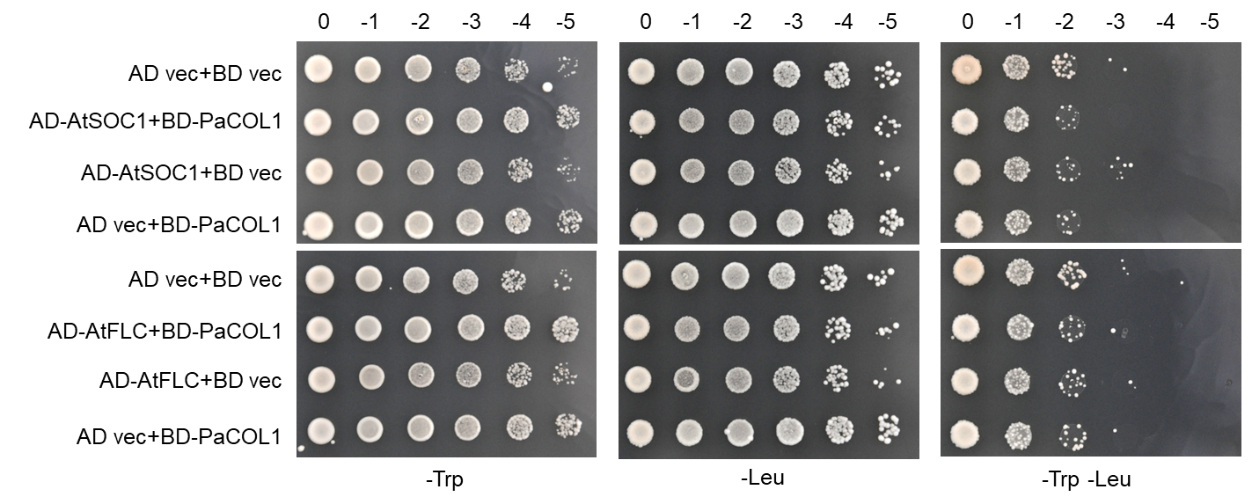


**Figure S5**: The negative control tests of AtSOC1 and AtFLC with PaCOL1 were grown on double dropout (-Trp -Leu) medium.


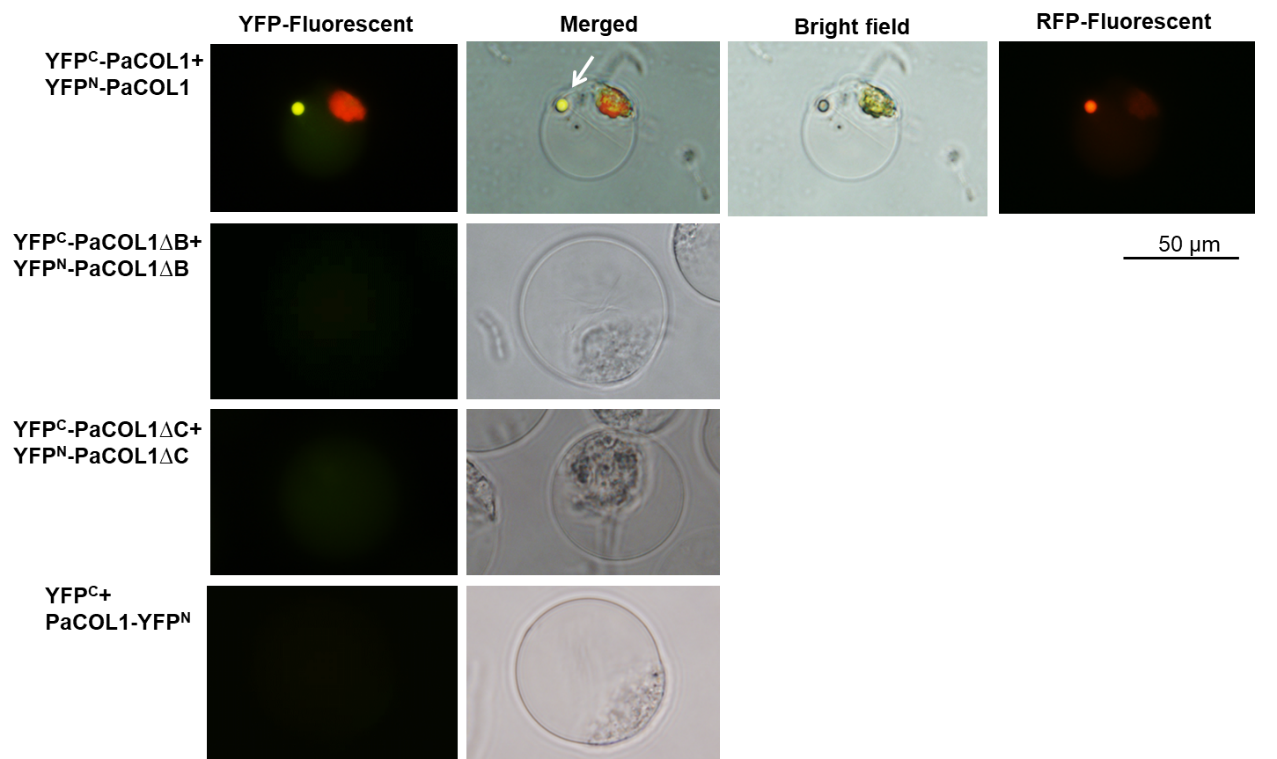


**Figure S6**: Interactions of PaCOL1, PaCOL1∆B and PaCOL1∆C in orchid petal protoplasts visualized by fluorescence microscopy. Orchid protoplasts were transformed with PaCOL1 and two truncated forms fused with half of YFP. YFP^C^ vector and PaCOL1-YFP^N^ were co-transformed as control. The arrow indicates cell nuclei. Scale bar = 50 µm.


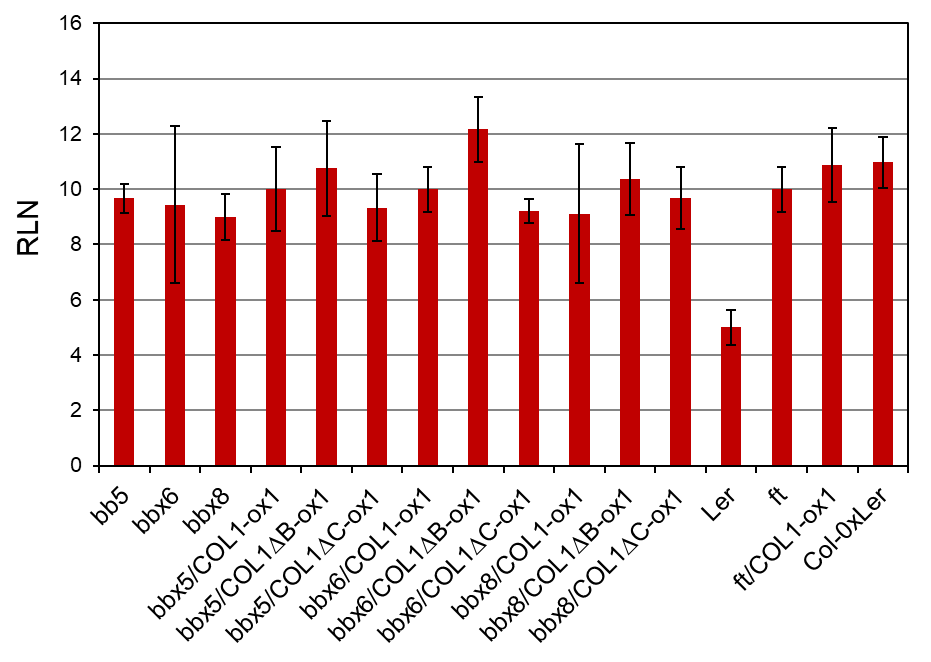


**Figure S7**: The complementary tests of PaCOL1, PaCOL1∆B and PaCOL1∆C over-expression in mutant lines under LD condition.


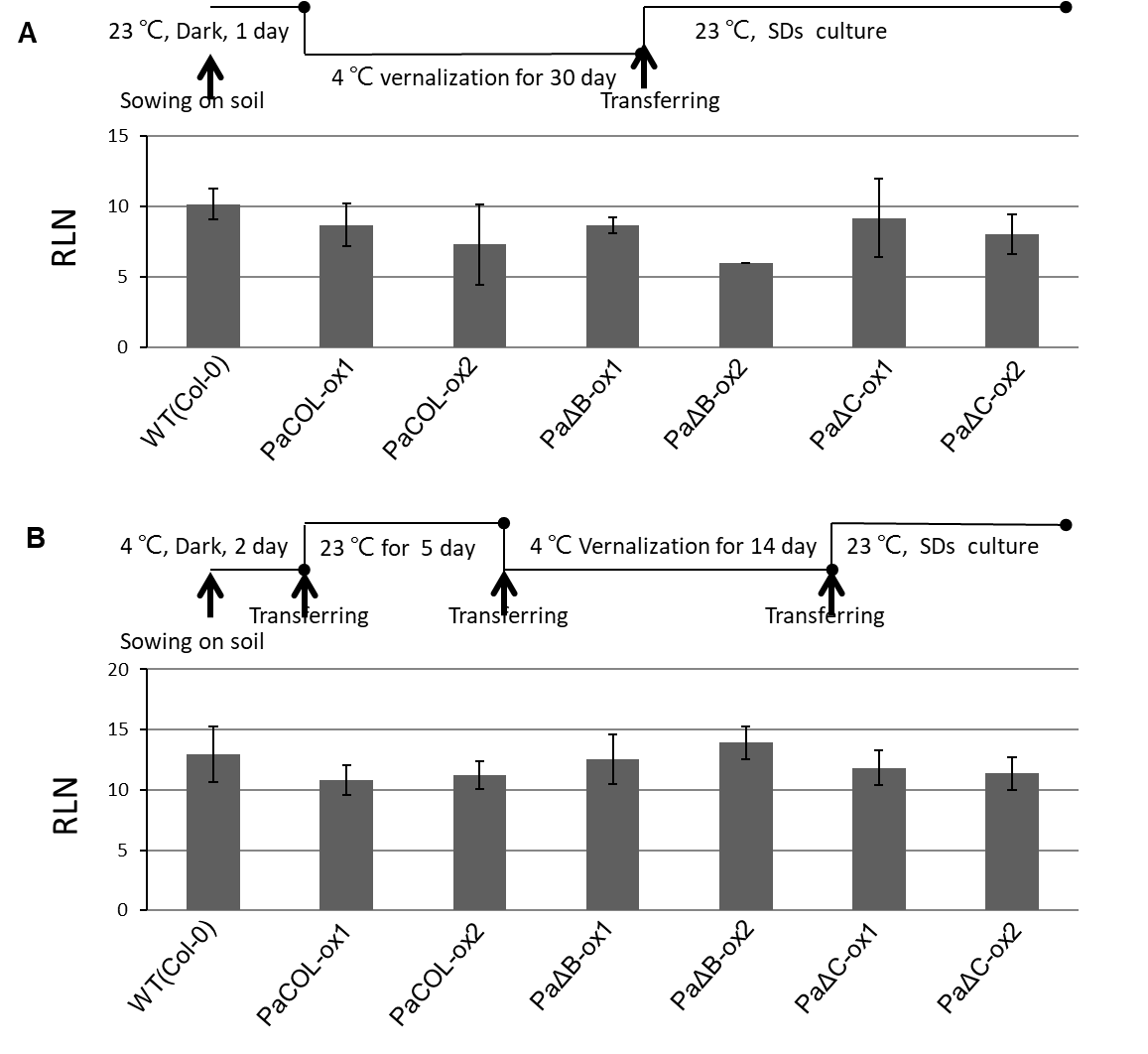


**Figure S8**: Cold treatment of transgenic *Arabidopsis* plants at seed imbibition and seedling stage.


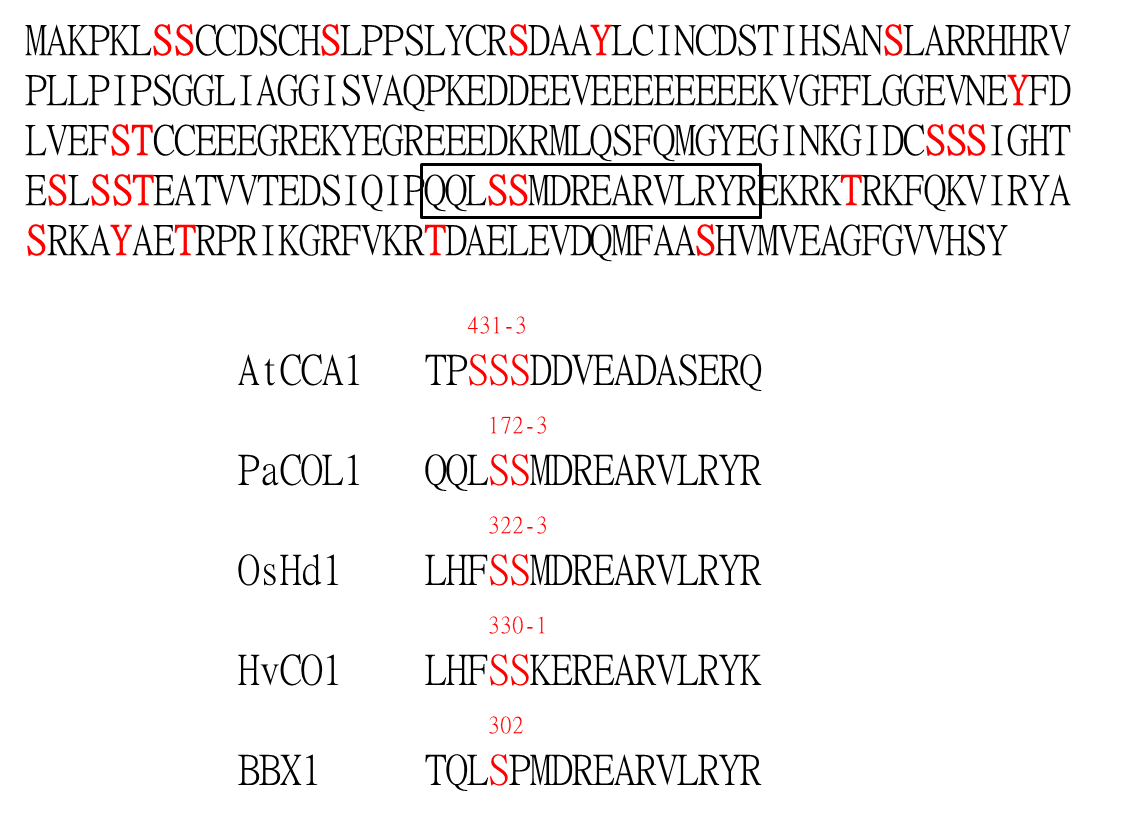


**Figure S9**: Phosphorylation site prediction of PaCOL1 and AtCCA1, OsHd1, HvCO1 and AtBBX1.
