# Supplementary material for: Molecular Characterization and Expression Profile of PaCOL1, a CONSTANS-like Gene in Phalaenopsis Orchid
Source: Plants (Basel). 2020 Jan 4;9(1):68. doi: 10.3390/plants9010068 (PMC7020484; doi:10.3390/plants9010068)
Supplement: Supplementary file 1 [file plants-09-00068-s001.zip › Yeh/Supplementary Table S2_103.docx]

Supplementary Table S2

Oligonucleotides used in this study.

| Gene | Primer | Sequence | Products (bps);  Use |
| --- | --- | --- | --- |
| AtAGL24 | PCR-Fw  PCR-Rv | 5’-GCGGCTGGAGAAACTACTTG-3’  5’-TCAAGGGGAGTTCCACTGTC-3’ | 239;  Gene expression |
| AtCCA1 | AD/BD-Fw  AD-Rv  BD-Rv | 5’-GGCATATGATGGAGACAAATTCG-3’  5’-ATCTCGAGTGTGGAAGCTTGAGT-3’  5’-ATGGATCCTGTGGAAGCTTGAGT-3’ | 1845;  Y2H |
|  | attB1-Fw  attB2-Rv | 5’-AAAAAGCAGGCTTCATGGAGACAAATTCG-3’  5’-AGAAAGCTGGGTCTGTGGAAGCTTGA-3’ | 1855;  BiFC |
|  | PCR-Fw  PCR-Rv | 5’-TGACCGGTCCTCGTGTGGCT-3’  5’-ACTGCGGCGTGCATTGGACT-3’ | 150;  Gene expression |
| AtCO | PCR-Fw  PCR-Rv | 5’-ATTCTGCAAACCCACTTGCT-3’  5’-CAACCTCCTTGGCATCCTTA-3’ | 183;  Gene expression |
| AtCDF1 | PCR-Fw  PCR-Rv | 5’- ACCCGGTTTACCCTTATTGG-3’  5’- TTGATCCCAAGTGTTGTCCA-3’ | 208;  Gene expression |
| AtFLC | AD/BD-Fw  AD-Rv  BD-Rv | 5’-CGCATATGATGGGAAGAAAAAAAC-3’  5’-ATCTCGAGATTAAGTAGTGGGAG-3’  5’-ATGGATCCATTAAGTAGTGGGAG-3’ | 607;  Y2H |
|  | attB1-Fw  attB2-Rv | 5’-AAAAAGCAGGCTCCATGGGAAGAAAA-3’  5’-AGAAAGCTGGGTCATTAAGTAGTGGG-3’ | 617;  BiFC |
|  | PCR-Fw  PCR-Rv | 5’- GACTGCCCTCTCCGTGACTA-3’  5’- TTCTCAACAAGCTTCAACATGAG-3’ | 69;  Gene expression |
| AtFT | PCR-Fw  PCR-Rv | 5’-GACCTCAGGAACTTCTATACTTTGGTTATG-3’  3’-CTGTTTGCCCTGCCAAGCTG-3’ | 219;  Gene expression |
| AtGI | PCR-Fw  PCR-Rv | 5’-GCTTGTGGAACTCCTTCGAG-3’  5’-TTCAATGGTTGCTTCTGCTG-3’ | 292;  Gene expression |
| AtMAF5 | PCR-Fw  PCR-Rv | 5’-CATGGAAGAGCAGCTCAAGA-3’  5’- CTTTCATCCCCTGTTTCCAA-3’ | 174;  Gene expression |
| AtNFYB2 | PCR-Fw  PCR-Rv | 5’-CGATTTGCTCTGGGCTATGACTA-3’  5’-CCTTCGATCTCCCTAAACCTCTG-3’ | 89;  Gene expression |
| AtNFYC3 | PCR-Fw  PCR-Rv | 5’-GCTGCGGTGACTAGAACTGA-3’  5’-ATTCCCGGGTTCCCAATTGG-3’ | 166;  Gene expression |
| AtSOC1 | AD/BD-Fw  AD-Rv  BD-Rv | 5’-ATCATATGATGGTGAGGGGCAA-3’  5’-CGCTCGAGCTTTCTTGAAGAAC-3’  5’-CGGGATCCCTTTCTTGAAGAAC-3’ | 658;  Y2H |
|  | attB1-Fw  attB2-Rv | 5’-AAAAAGCAGGCTCCATGGTGAGGGG-3’  5’-AGAAAGCTGGGTCCTTTCTTGAAGA-3’ | 668;  BiFC |
|  | PCR-Fw  PCR-Rv | 5’- AACAACTCGAAGCTTCTAAACGTAA-3’  5’- CCTCGATTGAGCATGTTCCT-3’ | 201;  Gene expression |
| AtSVP | AD/BD-Fw  AD-Rv  BD-Rv | 5’-GGCATATGATGGCGAGAGAAAA-3’  5’- ATCTCGAGACCACCATACGGTAA-3’  5’- ATGGATCCACCACCATACGGTAA-3’ | 736;  Y2H |
|  | attB1-Fw  attB2-Rv | 5’-AAAAAGCAGGCTCCATGGCGAGAGAA-3’  5’-AGAAAGCTGGGTCACCACCATACGGT-3’ | 746;  BiFC |
|  | PCR-Fw  PCR-Rv | 5’-AGAAGGCCCTTGAAACTGGT-3’  5’-CAAGTCGCTCGTTCTCTTCC-3’ | 162;  Gene expression |
| AtTFL1 | PCR-Fw  PCR-Rv | 5’- CAAGGCCAAGCATAGGGATA-3’  5’- GTGCAGCGGTTTCTCTTTGT-3’ | 183;  Gene expression |
| AtTSF | PCR-Fw  PCR-Rv | 5’-CACCACTGGAAATGCCTTTGGC-3’  5’-CGAGTTGCCGGAACAATACCAAC-3’ | 101;  Gene expression |
| AtTUB | PCR-Fw  PCR-Rv | 5’-CTCAAGAGGTTCTCAGCAGTA-3’  5’-TCACCTTCTTCATCCGCAGTT-3’ | 495;  Gene expression |
| PaACT9 | PCR-Fw  PCR-Rv | 5’- GGCTAACAGAGAGAAGATGACC-3’  5’- AATAGACCCTCCAATCCAGAC-3’ | 697;  Gene expression |
| PaCOL1 | Full-Fw  Full-Rv | 5’- ATCTAGAATGGCGAAGCCCAAAC-3’  5’- TTGGATCCCTAGTAAGAATGAACA-3’ | 759;  Transgenic line (COL1-ox), Gene expression |
|  | GFP-Fw  GFP-Rv | 5’- ATCTAGAGGATGGCGAAGCCCAAAC-3’  5’- TTGGATCCGGTAAGAATGAACA-3’ | 750,  Localization |
|  | ∆B-F  Full-Rv | 5’- ATCTAGAATGGCGAAGCCCCTCCCTA-3’  5’- TTGGATCCCTAGTAAGAATGAACA-3’ | 603;  Transgenic line (COL1∆B-ox), Gene expression |
|  | Full-Fw  PaCOL1∆C-R1  PaCOL1∆C-F2  Full-Rv | 5’- ATCTAGAATGGCGAAGCCCAAAC-3’  5’- CAAGTTCTGCTCTGTCCATGGAACTCAACT-3’  5’- CATGGACAGAGCAGAACTTGAAGTTGATCA-3’  5’- TTGGATCCCTAGTAAGAATGAACA-3’ | 630;  Transgenic line (COL1∆C-ox), Gene expression |
|  | 376BamHI-Fw  744BamHI-Rv | 5’- AGGATCCATGTTGCAGAGTTTTCA-3’  5’- AGGATCCCTAGTAAGAATGAAC-3’ | 368;  Antibody production |
|  | AD/BD-Fw  AD/BD-Rv | 5’-GGAGGCCGAATTCATGGCGAAGCCCAAACTCAGTTCC-3’  5’-GGTCGACGGATCCGTAAGAATGAACAACACCAAAGCC-3’ | 772;  Y2H |
|  | attB1-Fw  attB2-Rv | 5’- AAAAAGCAGGCTTCATGGCGAAGCCC-3’  5’- AGAAAGCTGGGTCGTAAGAATGAACA-3’ | 755;  BiFC |
|  | PCR-Fw  PCR-Rv | 5’- CTTTCTTGGAGGTGAGGTGA-3’  5’- ACTCTGCAACATCCGCTTAT-3’ | 121;  Gene expression |

The primers were designed according to the Primer3 software (version 0.4.0, http://primer3.sourceforge.net/releases.php).
